# Robotic-assisted surgery for esophageal submucosal tumors: a single-center case series

**DOI:** 10.1007/s13304-022-01247-z

**Published:** 2022-02-11

**Authors:** Caterina Froiio, Felix Berlth, Giovanni Capovilla, Evangelos Tagkalos, Edin Hadzijusufovic, Carolina Mann, Hauke Lang, Peter Philipp Grimminger

**Affiliations:** 1 Mainz University, Johannes Gutenberg Universitat Mainz, Mainz, Germany; 2 Department of General Surgery , IRCCS Policlinico San Donato, University of Milan , Milano, Italy

**Keywords:** Esophageal submucosal tumor, SMTs, Robotic enucleation, Robotic-assisted minimally invasive esophagectomy

## Abstract

Esophageal submucosal tumors (SMTs) are rare heterogenous clinical entities. The surgical resection can be performed in different surgical approaches. However, the robotic surgical strategy is poorly documented in the treatment of SMTs. We present our series of operated esophageal SMTs approached via robotic-assisted surgery. Six patients with symptomatic esophageal submucosal tumors underwent robotic surgery within a 3-year period. The performed procedures were robotic-assisted enucleation, robotic esophagectomy (RAMIE) and reverse hybrid robotic esophagectomy. Patients’ clinical data, intra/postoperative outcomes, and histopathological features were retrieved from the institution’s prospective database. Five of six patients were scheduled for upfront surgery: four underwent robotic enucleation (three leiomyoma and one suspected GIST) and one underwent reverse hybrid robotic esophagectomy (suspected GIST). One patient, diagnosed with GIST, was treated with neoadjuvant Imatinib therapy, before undergoing a RAMIE. No major intra-operative complications were recorded. Median length of stay was 7 days (6–50), with a longer post-operative course in patients who underwent esophagectomy. Clavien–Dindo > 3a complications occurred in two patients, aspiration pneumonia and delayed gastric emptying. The final histopathological and immuno-histochemical diagnosis were leiomyoma, well-differentiated GIST, low-grade fibromyxoid sarcoma and Schwannoma. Robotic-assisted surgery seems to be a promising option for surgical treatment strategies of benign or borderline esophageal submucosal tumors.

## Introduction

Esophageal submucosal tumors (SMTs) are rare, accounting for less than 1% of all esophageal neoplasms [1]. They arise from mesenchymal, muscle, vascular, nerve or glandular cells, defining a histopathological heterogeneous group of tumors with different clinical implications. Despite being more frequently benign lesions (e.g., leiomyoma, lipoma), they may also show borderline or malignant clinical behavior (e.g., GIST, sarcoma) [2–4]. If symptomatic, the most frequent clinical manifestation is dysphagia, related to the size of the tumor.

Neither endoscopic nor radiological imaging techniques can always deliver an accurate clinical differential diagnosis between benign and malignant lesions, and the pre-operative histopathological definition is not always conclusive [5]. This challenge, combined with the low incidence of SMTs leads to a non-standardized management in terms of diagnostic and therapeutic treatment pathways.

Surgical resection of esophageal submucosal tumors (enucleation, resection up to subtotal esophagectomy), represents the mainstay in the treatment of suspected malignant tumors and of large symptomatic lesions [6–8].

Minimally invasive surgery including a thoracoscopic surgical approach is established in the treatment of esophageal diseases and offers the advantages of a shorter hospitalization and a faster recovery, compared to open surgery. A robotic-assisted approach, with high‐definition 3‐d vision and a 7‐degree articulation of the instruments could significantly improve the surgeon’s ability to manage difficult anatomical features in a narrow space such as the thoracic cavity, overcoming the weakness of thoracoscopy in the management of complex cases.

In this case series, we present a series of submucosal esophageal tumor approached with different techniques via robotic-assisted esophageal surgery.

## Materials and methods

We conducted a retrospective review of all consecutive patients referred to our Institute for SMTs of the esophagus, including both benign and borderline tumors, that underwent robotic-assisted surgical treatment.

Patient’s preoperative data, intraoperative and post-operative outcomes were retrieved from the institution`s prospective database. Demographic and clinical data included sex, age, BMI, ASA score, Charlson Comorbidity Index, clinical presentation, location and size of the lesions, and preoperative histologic features.

Diagnostic preoperative work-up included flexible endoscopy, endoscopic ultrasound-guided fine-needle aspiration (EUS-FNA), and chest and abdominal contrast-enhanced computed tomography (CT-Scan). The positron emission tomography (PET-CT) was performed only in patients with clinical suspicious of malignant tumors. Indication for surgery included the preoperative diagnosis of GIST in three cases (50%) and the symptomatic increasing tumor size in the other cases. One patient with the preoperative diagnosis of GIST, requiring multi-modal treatment, was treated with neoadjuvant imatinib chemotherapy, according to final decision of multidisciplinary tumor board.

Post-operative complications were graded according to the Clavien–Dindo classification [9]. Histological and immuno-histochemical characteristics of the tumors were also reviewed.

Continuous variables were described as mean (range) and median (IQR), while categorical variables were described using frequencies and percentages.

All procedures were conducted in accordance with the ethical standards and with the Helsinki Declaration. Informed consent was not required due to the retrospective design of the study.

## Surgical technique

### Surgical position

All procedures were performed with DaVinci Xi^®^ System (Intuitive Surgical Inc. Sunnyvale, CA, USA). A double-lumen endotracheal tube was used to obtain single-lung ventilation for the thoracic phase. The patients were placed in a left lateral semi-prone position (with the‬ operation table tilted 30° toward prone) and patient’s right arm was raised cranially. The DaVinci Xi^®^ System was placed on the patient’s right side and the assistant surgeon was located on the patient’s left side [10].‬‬‬‬‬‬‬‬‬‬

### Robotic enucleation

**Fig. 1:**
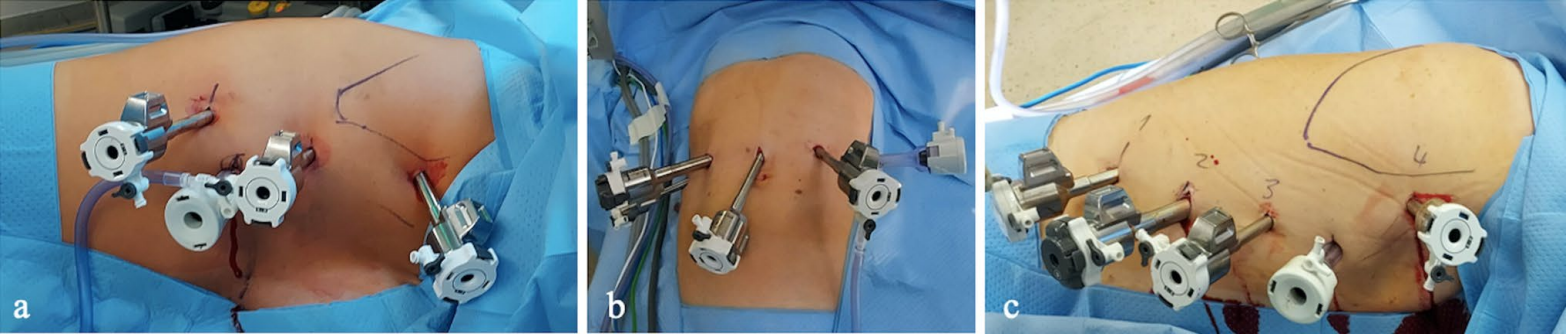
**a** Trocar placement for robotic enucleation; **b** and trocar placement for abdominal part and thoracic part in RAMIE

The three robotic trocars were placed in the following positions (Fig. 1a): the 8 mm camera trocar along the posterior axillary line in 6th intercostal space (ICS); the 8 mm “right-hand” trocar at the posterior axillary line in 4th ICS; the 8 mm “left-hand” trocar in posterior axillary line in 8th ICS; and an assistant laparoscopic 12 mm trocar was placed along the anterior axillary line in the 7th ICS. A mild pneumothorax was induced using a pressure of 7 mmHg. The DaVinci Xi^®^ System docking was then performed.

After diagnostic thoracoscopy using the DaVinci Xi 8 mm 30° Endoscope, we opened the mediastinal pleura up to 2 cm above the azygos vein to expose the thoracic esophagus. The placement of a 38 Ch gastric tube was useful to define the extent of the tumor and to preserve the opposite wall of the esophagus. The serosa and the muscular layer were opened by the Permanent Monopolar Cautery Hook placed in right-hand trocar. Subsequent careful enucleation of the tumor was performed through cautious dissection, to protect the mucosa and to avoid the rupture of the lesion. The Bipolar Fenestrated Forceps in the left-hand were used as a retractor. In one patient, the opening of the esophageal mucosa occurred, due to the tenacious adhesion between the mucosa and the muscular layer in the site of a previous EUS-FNA, and it was closed using a single PDS 4/0 stich. After recovering the specimen in a bag, the muscular and serosal layers were then approximated with two running V-loc 4/0 sutures (V-Loc™, Covidien, Mansfield, MA). The esophagus was covered with a pleural tent fixed with V-loc 4/0 or PDS 4/0 running suture. Hemostasis was conducted, when necessary, using the bipolar forceps or the monopolar hook. After undocking the DaVinci Xi^®^ System, the specimen could be extracted through an enlargement of the assistant 12 mm trocar. A 20 Ch chest tube was left in the pleural space through the left-hand 8 mm trocar, the re-expansion of the lung was controlled under vision.

### Robotic sub-total esophagectomy

The surgical procedure was previously extensively described by our group [10, 11]. We describe it briefly below.

#### Abdominal part

For the abdominal part, the patient was in a supine position, and the trocars were placed in a horizontal line at or above the umbilicus as follows (Fig. 1b): an 8 mm robotic trocar in the right mid-abdomen (trocar 1); a 12 mm robotic trocar between the umbilicus and trocar 1 (trocar 2, “left-hand trocar”); an 8 mm camera robotic trocar at or above the umbilicus (trocar 3); an 8 mm robotic trocar placed 6–8 cm laterally to the robotic umbilical trocar (trocar 4, “right-hand” trocar). One 12 mm assistant trocar was positioned at the upper left abdominal quadrant. In the abdomen, CO_2_ insufflation with a 14 mmHg was used. The DaVinci Xi^®^ System docking was then performed. The da Vinci Xi 8 mm 30° Endoscope was placed in the trocar 3. The mobilization of the stomach was performed using the Vessel Sealer Extend™ or SynchroSeal™ (Intuitive, Sunnyvale, CA) in the trocar 4, and the Fenestrated Bipolar Forceps (trocar 2) or Tip-up Fenestrated Grasper (trocar 1) was used for retraction; this last one was also used to lift the left lobe of the liver. The creation of a gastric conduit was performed using the robotic SureForm^®^ 60 mm stapler (in 12 mm trocars) starting at the lesser curvature near the crow foot and proceeding with another two 60-mm blue magazines toward the fundus, parallel to the greater curvature; 6 to 7 cm of stomach were left to complete the gastric conduit in the thoracic part. A Permanent Monopolar Cautery Hook was used for the lymph node dissection (trocar 4), and the EndoWrist^®^ Clip Applier was used to clip the left gastric artery. The assistant trocar was used for suction, retraction, and collection of resected specimens, as lymph nodes.

#### Thoracic part

Patient position, DaVinci Xi^®^ System and assistant surgeon settings were the same described for robotic enucleation.

Trocars were placed as follows (Fig. 1c): the 8 mm camera trocar was placed between the middle and the anterior axillary line in the 6th ICS; the “right-hand” 8 mm trocar was placed along the posterior axillary line in the 4th ICS and the “left-hand” 12 mm trocar was placed along the middle axillary line in the 8th ICS; an 8 mm trocar was placed in the 10th ICS along the posterior axillary line for retraction; a 12 mm laparoscopic assistant trocar was placed in the 5th ICS along the anterior axillary line. The dissection of the esophagus and the thoracic 2-Field lymphadenectomy were performed using the Permanent Monopolar Cautery Hook. If needed, the azygos vein was clipped using the EndoWrist^®^ Clip Applier through trocar 4 and transected with the Vessel Sealer. The esophagus was transected above the azygos vein using the monopolar hook. The purse string suture in the esophageal stump was performed robotically using a 90 cm Prolene 2/0 (Ethicon, USA). Next, the DaVinci Xi system was temporary undocked. The 12 mm assistant trocar incision was widened to create a mini-thoracotomy, and an Alexis O Wound Retractor (Alexis™ Laparoscopic System, Applied Medical) was inserted. The thoracotomy was then used to introduce the anvil of the circular stapler (28 mm EEA™, Covidien, Mansfield, MA, USA) and inserted it in the esophageal stump. The specimen was pulled toward the mediastinum, exteriorized through the mini-thoracotomy, and opened to insert the circular stapler into the stomach. The stapler was then pushed into the chest and the circular anastomosis was performed. The da DaVinci Xi^®^ System was docked again to complete the final step in stapling the rest of the gastric conduit with the 60 mm robotic SureForm^®^ stapler; a thick 38-ch gastric tube was introduced into the gastric conduit to prevent its narrowing. Then, the specimen was completely stapled and retrieved. We usually reinforce the circular anastomosis with 2 hemi-circumferential running sutures, using 2 barbed monofilament absorbable stitches (V-Loc™, Covidien, Mansfield, MA). Hemostasis was conducted, when necessary, using the bipolar forceps or the energy device (Vessel Sealer Extend™ or SynchroSeal™, Intuitive, Sunnyvale, CA). One chest drain was inserted via the robotic trocar at ICS 10 after undocking the robot.

### Reverse Robotic Hybrid esophagectomy

This surgical procedure consisted of a primary thoracic robotic phase, a subsequent abdominal laparotomy phase for the resection of the large tumor mass (see CT scan Fig. 3), followed by the robotic thoracic phase to perform the Ivor Lewis esophagectomy and intrathoracic esophagogastric anastomosis.

The thoracic phase was described above: with the patient in a left semi-prone position and single-lung ventilation, the da DaVinci Xi^®^ System was docked. After mobilizing the esophagus, a standard thoracic lymphadenectomy was performed, the esophagus was stapled using EndoGia™ stapler (45 mm purple, Covidien, Mansfield, MA, USA). The azygos vein and the thoracic duct were preserved.

The patient was then placed in a supine position. Through a median supra-umbilical laparotomy, the gastro-lysis and standard lymphadenectomy were carried out. The gastric conduit was created using an EndoGia™ stapler (45 mm purple, Covidien, Mansfield, MA, USA), starting at the lesser curvature toward the fundus, parallel to the greater curvature. In this case, the conduit was completely separated from the remaining portion of the stomach. The specimen could thus be retrieved through the diaphragmatic hiatus and be sent for definitive histological examination. The gastric conduit was then transposed in the mediastinum, and a posterior hiatoplasty was performed.

The patient was repositioned on the semi-prone left side with a mild pneumothorax. The esophagus was opened at the level of the previous suturing line and, through a 4 cm mini-thoracotomy along the 12 mm assistant trocar, the anvil of a circular stapler (28 mm EEA ™, Covidien, Mansfield, MA, USA) was inserted into the esophageal stump. After choosing the site for the anastomosis, at the greater curvature, the intrathoracic circular-stapled anastomosis with reinforcement of the pleural flap was performed, as described above. The remaining part of the stomach was stapled with an EndoGia™ stapler (60 mm purple, Covidien, Mansfield, MA, USA) and retrieved. A 20 Ch chest drain was placed in the pleural space through the lowest left 8-mm trocar.

## Results

**Fig. 2:**
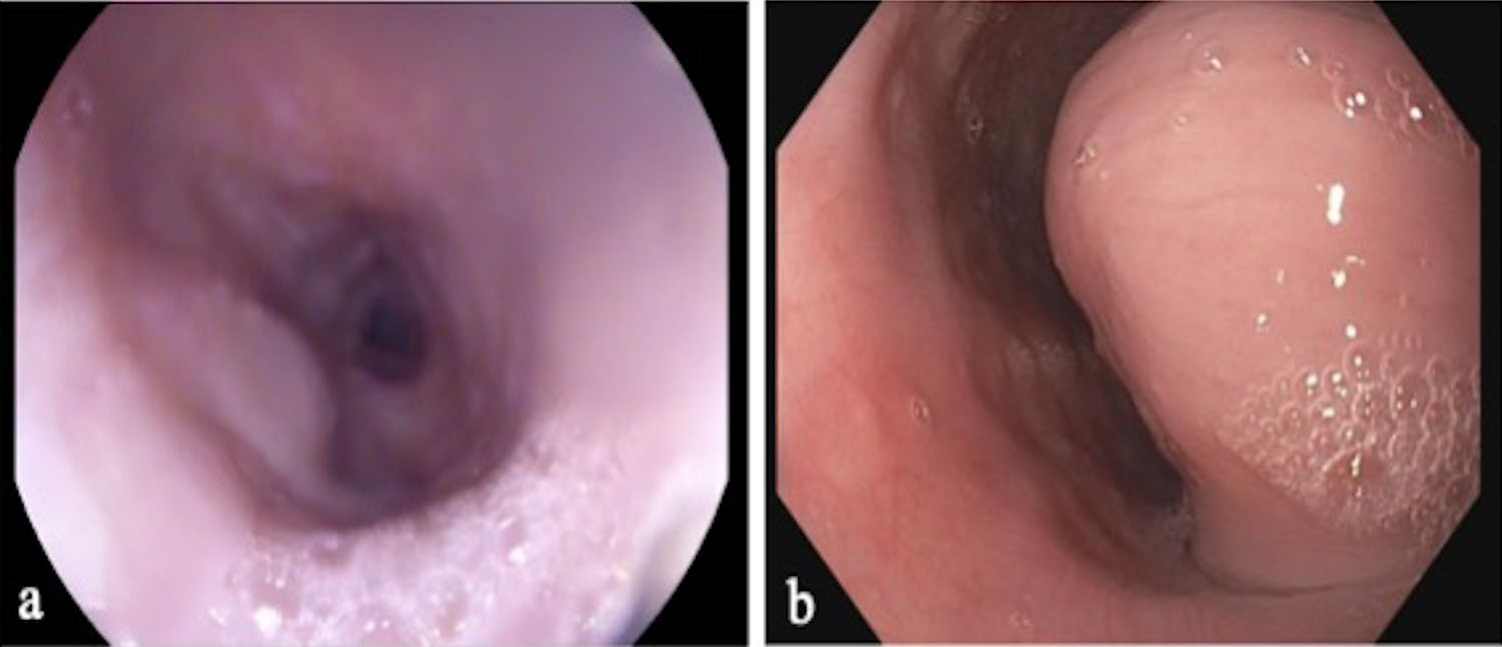
Endoscopic view of **a** GIST and **b** Schwannoma

**Fig. 3:**
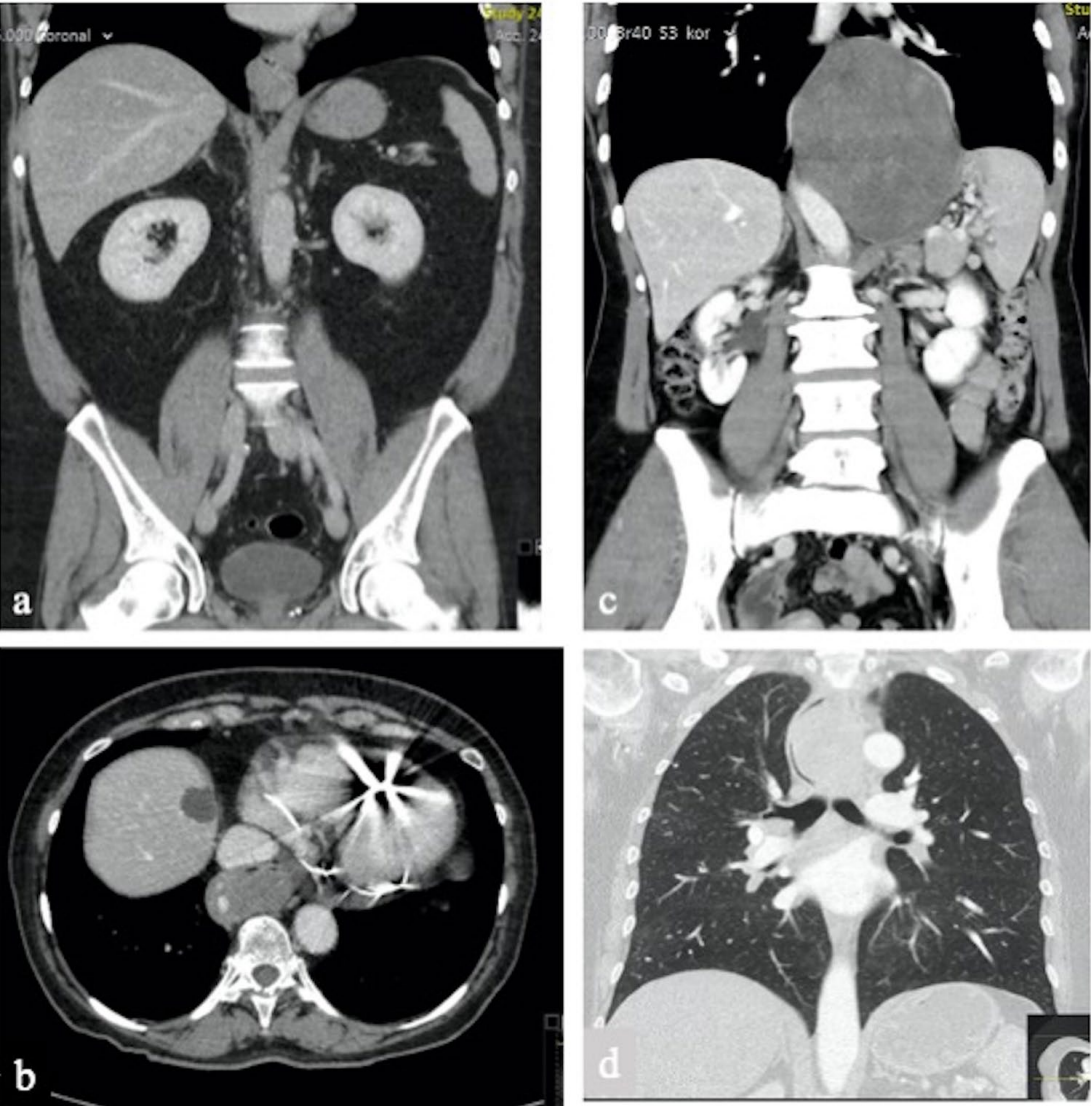
**a** leiomyoma of the distal esophagus; **b** GIST of the distal esophagus; **c** low-grade fibromyxoid sarcoma of the distal esophagus; **d** Schwannoma of the proximal esophagus

Six patients with esophageal submucosal tumors were robotically operated over a 3-year period. The preoperative and clinical data, including concomitant tumor’s characteristics and indication to surgery, are summarized in Table 1. The mean age was 57.3 years (range 50–77) and male to female ratio was 1:5. The most frequent clinical presentation was dysphagia (100%), other referred symptoms were weight loss (2 of 6 patients; 33.3%), chest discomfort (1 of 6 patients; 16.7%) and regurgitation (1 of 6 patients; 16.7%). The diagnosis of a submucosal esophageal tumor was made through endoscopy in all patients (Fig. 2). The lesions were distributed at the proximal esophagus (1 of 6 patients; 16.7%), at the middle esophagus (2 of 6 patients; 33.3%) and at the distal esophagus (3 of 6 patients; 50%). All patients underwent chest–abdominal contrast-enhanced CT scan (Fig. 3). Two patients with suspicious malignant lesions underwent PET-CT, that in both cases did not show specific FDG avidity. No distant lesions suspected of malignancy were found in any patient. Ultrasound endoscopy was performed in all patients, confirming the submucosal origin of the tumors, and a Fine-Needle Aspiration (EUS-FNA) for cytochemical diagnosis was performed. The preoperative histologic diagnosis was leiomyoma in three patients (50%), GIST in two patients (33.3%) and GIST Wilde Type in one patient (16.7%).

**Table 1:** Clinical and pre-operative data

Patient	Age	Sex	ASA	CCI	BMI	Clinical presentation	Diagnostic procedures	Site of the tumor	Tumor size (cm)	Preoperative diagnosis	Neoadjuvant therapy	Indication to surgery
1	50	M	2	3	31.14	Dysphagia	EGDS CT-SCAN EUS-FNA	Distal esophagus	5.0 × 4.5x3	Leiomyoma	**–**	Increasing tumor size; symptomatic tumor
2	77	F	3	7	27.7	Dysphagia Weight loss	EGDS CT-SCAN PET-CT EUS-FNA	Distal esophagus	7.7 × 5x10.6	GIST	Imatinib	Planned surgery post CT (partial response)
3	55	F	2	4	37.77	Dysphagia Regurgitation	EGDS CT-SCAN EUS-FNA	Middle esophagus	2.8 × 1.5	Leiomyoma	–	Symptomatic tumor
4	54	F	2	4	36.67	Dysphagia	EGDS CT-SCAN EUS-FNA	Middle esophagus	4 × 3x2.5	Leiomyoma	–	Increasing tumor size; symptomatic tumor
5	55	F	2	4	20.86	Dysphagia Weight loss Chest discomfort	EGDS CT-SCAN PET-CT EUS-FNA	Distal esophagus/cardias	9.6 × 9.2x8	GIST Wilde Type	No	Histology
6	55	F	2	4	29	Dysphagia	EGDS CT-SCAN EUS-FNA	Proximal esophagus	6.5 × 4.7	GIST	No	Histology

*CCI* Charlson Comorbidity Index; *BMI* Body Max Index; *GIST* Gastrointestinal Stromal Tumor

**Fig. 4:**
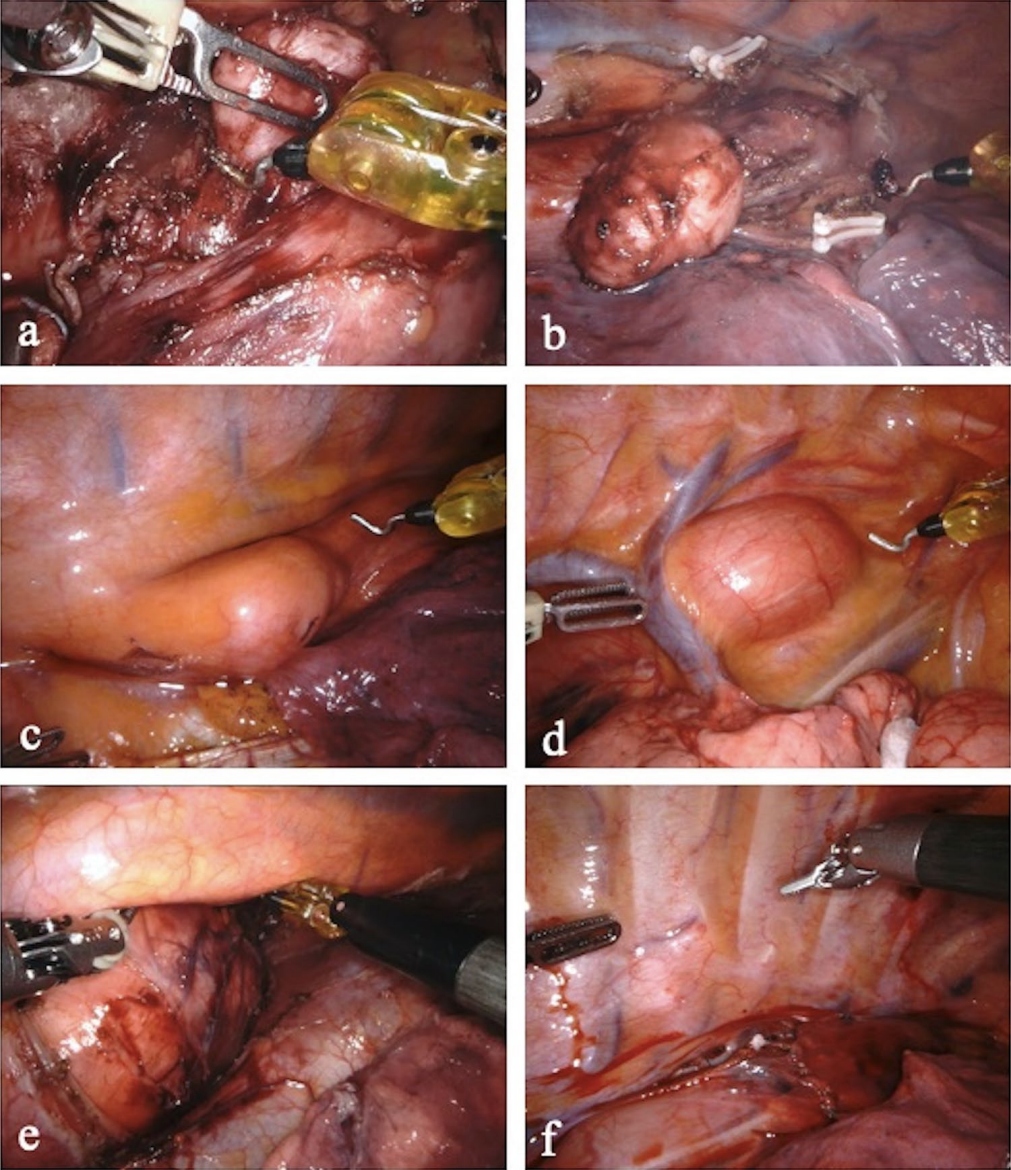
Intraoperative findings: **a** and **b** enucleation of a leiomyoma of the middle esophagus, (the azygos vein is sectioned between Hem-o-Lock); **c** leiomyoma of the distal esophagus; **d** Schwannoma of the proximal esophagus; **e** giant low-grade fibromyxoid sarcoma of the distal esophagus; **f** the esophagus is covered with a pleural tent fixed with V-loc 4.0 running suture

Among patients diagnosed with GIST, two underwent upfront surgery and one patient, according to the final decisions of multidisciplinary tumor board, was referred to a neoadjuvant treatment with Imatinib. The subsequently revaluation resulted in a partial regression of the tumor and the patient was then scheduled for surgery. The choice of surgical strategies took into account the location of the tumor, the preoperative histological diagnosis and the size of the lesion. The surgical procedures included robotic enucleation (leiomyoma or small GIST), robotic Ivor Lewis esophagectomy (RAMIE) with 2-Field lymphadenectomy (GIST); and hybrid robotic reverse esophagectomy with a 2-Field lymphadenectomy (robotic thorax phase and laparotomic abdominal phase; suspected GIST), due to the large size of the lesion involving the abdominal cavity. The Fig. 4 shows the intraoperative findings.

Intra-operative and post-operative outcomes are reported in Table 2. All the procedures were completed by the planned robotic approach, without conversion. No major intraoperative complications, such us bleeding, airway injuries, vascular injury, inability to maintain single-lung ventilation, occurred. We recorded only one case of esophageal mucosa opening, due to previous biopsy. The dissection of tumors was carried out en bloc, maintaining the capsule integrity in all cases. The median duration of surgery was 154.5 min.

**Table 2:** Intraoperative and postoperative outcomes

Patient	Operation type	Operative time (min)	Opening mucosa	Intraoperative complication	Conversion	C-D > 3	LOS (days)	ICU (days)	Inhospital mortality	FU (months)	Recurrence
1	Robotic enucleation	129	No	No	No	None	7	0	0	24	No
2	Robotic esophagectomy	311	-	No	No	Yes*	50	20	0	14	No
3	Robotic enucleation	111	No	No	No	None	7	0	0	14	No
4	Robotic enucleation	127	Yes	No	No	None	6	0	0	14	No
5	Reverse hibryd robotic esophagectomy	249	-	No	No	Yes**	11	1	0	12	No
6	Robotic enucleation	180	No	No	No	None	6	0	0	2	No
Total *n* = 6		Median (range)	*n* (%)	*n* (%)	*n* (%)	*n* (%)	Median (range)	Mean (range)	*n* (%)	Mean (range)	*n* (%)
		154.5 (129–232)	1 (16.7)	0 (0)	0 (0)	2 (33.3)	7 (6–50)	3.5 (0–20)	0 (0)	13.4 (2–24)	0

*C–D* Clavien–Dindo complication grade; *LOS* Length of Stay; *ICU* Intensive Care Unit stay; *FU* Follow-Up

*C–D 4a = Aspiration pneumonia

**C–D 3a = Delayed Gastric Empty

We observed two cases of postoperative Clavien–Dindo > 3a complications: one patient suffered from aspiration pneumonia and subsequent respiratory failure, that required reintubation and referral to the intensive care unit; one patient experienced a delayed gastric empty treated by endoscopic dilatation of the pylorus.

The median postoperative length of stay was 7 days (6–50). There was no in-hospital mortality. At a mean follow-up of 13.4 months (2–24), all patients were alive with no detectable recurrence.

Definitive postoperative histopathologic and immuno-histochemical characterization confirmed the pre-operative diagnosis of leiomyoma in all cases. Among patients with preoperative diagnosis of GIST, confirmation was only found in one case, while in the other two cases, the final diagnoses were low-grade fibromyxoid sarcoma and Schwannoma (Table 3). All procedures were carried out as R0 resection.

**Table 3:** Immuno-histopathological staining and pathological diagnosis of the tumor

Patient	c-Kit	DOG-1	Desmin	S-100	SMA	CD34	ALK-1	STAT-6	Ki-67	Mitosis/50 HPF	Definitive diagnosis
1		−	+	nt	+	nt	nt	nt	2% (MIB−1)	−	Leiomyoma
2	+	+	nt	nt	nt	nt	nt	nt	10%	2	GIST EXON-9 mutation*
3	−	−	+	nt	+	nt	nt	nt	< 1%	−	Leiomyoma
4	−	−	+	nt	+	−	nt	nt	< 1%	−	Leiomyoma
5	−	−	−	−	−	−	−	−	1–2%	2	Low-grade fibromyxoid sarcoma**
6	−	−	−	+	−	−	−	nt	5%	1	Schwannoma**

*nt* not tested

*Molecular analysis by PCR (polymerase chain reaction)

**Definitive diagnosis differs from the preoperative one

## Discussion

Since the low incidence of the esophageal SMTs [1–3], a standardization in their clinical management is difficult.

We reported our case series of esophageal SMTs approached via robotic-assisted surgery, describing the surgical technical details, the intraoperative and post-operative outcomes. Considering the high morbidity and mortality associated to esophageal surgery [12, 13], an adequate diagnostic path is mandatory to select patients for primary surgery and/or neoadjuvant therapy, and for the choice of the most suitable surgical treatment (enucleation vs major resection).

The diagnostic work-up is based on endoscopy and endo-sonographic features [3, 5, 14] and radiological investigation as CT scan with contrast enhancement and FDG-PET [15, 16]. The role of preoperative histology with EUS-FNA or cut biopsy is still on debate; biopsies are often inconclusive or inconsistent with final pathological examinations. Furthermore, the procedure is burdened by the risk of tumor dissemination through a capsule interruption and may cause esophageal mucosal damage with subsequently difficult identification of planes during surgical enucleation [7, 8, 17]. In our series, two of three patients with preoperative diagnosis of GIST resulted in an alternative definitive pathological finding. Furthermore, previous EUS-FNA resulted in the opening of the mucosal layer in one patient.

The choice of the type of operation, enucleation or esophagectomy, depends on the localization, size and suspected malignant behavior of the tumor [5, 18, 19]. Enucleation is widely accepted in case of benign lesions (cysts, leiomyomas) or GIST of less than 5 cm [8, 20], while esophagectomy is preferred as the treatment of choice for larger tumors, localized in challenging positions or highly suspicious for malignancy, such as the presented case no. 5 (Table 1) [18, 19].

The minimally invasive approach as thoracoscopy has brought important advantages in esophageal surgery, such as less blood loss, faster postoperative stay, a shorter recovery and a lower rate of perioperative complications, becoming the preferred surgical approach for such lesions [4, 13, 21].

Since the first thoracoscopic enucleation of an esophageal leiomyoma, described by Everitt in 1992 [22], this approach has been increasingly chosen from several Authors, who described its feasibility and safety in the treatment of leiomyomas or GISTs [4, 23, 24]. Similarly, prone thoracoscopic esophagectomy has gained acceptance for the treatment of challenging submucosal tumors, characterized by large size or malignant behavior, with favorable short-term and oncological outcomes [25–27].

The advancement of robotic technologies has made it possible to overcome the technical limitations of laparoscopic or thoracoscopic surgery, such as the lack of intra-corporeal articulation of the surgical instruments, and the poor ergonomics of the surgeon in performing complex procedures [28]. The robotic system facilitates technically difficult dissections, sutures, and intrathoracic preparation and may prevent esophageal injuries or tumors’ rupture during enucleations. The safety and feasibility of the robotic approach for esophageal cancers have been previously described, [29, 30] and the intraoperative and postoperative benefits can reasonably be exploited in the treatment of these borderline tumors.

In literature, the safety and feasibility of the robotic approach of mesenchymal tumors has recently been described for the treatment of gastric GIST in unfavorable locations by Arseneaux [31] and Winder [32], showing optimal surgical and oncological outcomes. However, although with satisfactory results, the published experiences concerning the robotic approach to esophageal SMTs are limited to some case reports and only one case series (Table 4). Since the first case of robotic enucleation of two esophageal leiomyomas was described, by Elli [33] in 2004, only few case reports have been reported in the past 15 years, all involving robotic treatment of esophageal leiomyomas [34–41] and one case of Schwannoma [42]. The only case series concerning the treatment of different esophageal SMTs (GIST, leiomyoma and cyst) was recently reported by Tribuzi et al., and the surgical approach consisted only of robotic enucleation [43].

**Table 4:** Previous published experiences in robotic approach to esophageal SMTs

Author year	No of patient	Histology	Site of the tumor	Tumor size (cm)	Operation Type	Operative time (min)	Opening mucosa	Conversion	LOS (days)	Overall reported complication	FU^⊥^ (months)
Elli et al. 2004 [33]	1	Leiomyoma	Proximal esophagus	5 × 3	Robotic enucleation	n.a	0	0	n.a	0	6
Bodner et al. 2005 [34]	2	Leiomyoma (1) Cyst (1)	Distal esophagus	2	Robotic enucleation	121 (147–95)*	0	0	7	0	5
DeUgarte et al. 2008 [35]	1	Leiomyoma	Middle esophagus	7 × 5	Robotic enucleation	n.a	0	0	5	0	24
Boone J et al. 2008 [36]	1	Leiomyoma	Proximal esophagus	9 × 5	Robotic reverse hybrid esophagectomy	270	-	0	11	0	36
Kerstintine et al. 2009 [37]	1	Leiomyoma	Distal esophagus	4 × 2.5	Robotic enucleation	104	0	0	5	0	36
Chiu et al. 2011 [38]	1	Leiomyoma	Proximal esophagus	2	Robotic enucleation	n.a	0	0	6	0	6
Khalaileh et al. 2013 [39]	1	Leiomyoma	Distal esophagus	4 × 3	Robotic enucleation	288	0	0	5.7	0	n.a
Compean et al. 2014 [40]	1	Leiomyoma	Middle esophagus	10 × 3	Robotic enucleation	n.a	0	0	4	0	n.a
Inderhees et al. 2019 [41]	1	Leiomyoma	n.a	6.5	Robotic enucleation	143	0	0	5	0	n.a
Zhang et al. 2018 [42]	1	Schwannoma	Middle esophagus	7 × 4	Robotic enucleation	108	0	0	5	0	50
Tribuzi et al. 2020 [43]	5	GIST (2) Leiomyoma (2) Cyst (1)	Lower esophagus (2) Middle esophagus (3)	3.7 (3–6.3)*	Robotic enucleation	150 (100–300)*	0	0	5 (4–9)*	0	16**

*LOS* Length of Stay; *FU* Follow-up; *n.a.* not available. ^⊥^ No recurrences were detected at follow-up

*Median and range

**Mean

To the best of our knowledge, this is the first series of robot-assisted surgery cases describing the application of several possible approaches (enucleation, RAMIE, Reverse-Hybrid RAMIE) for esophageal submucosal tumors. Furthermore, we report a heterogenous group of esophageal SMTs, with different clinical behavior, successfully treated regardless of the size, esophageal site and anatomical complexity related to some of them. Our experience of robotic-assisted surgery for submucosal esophageal tumors resulted in excellent surgical outcomes without intraoperative complications or need for conversion. In only one patient, we planned a hybrid approach due to the large size of the tumor (Fig. 3c) and its localization. The onset of a mucosal lesion during the enucleation of a voluminous leiomyoma was adequately managed with the help of the precise and articulated robotic instruments. The reported post-operative complications, aspiration pneumonia and delayed gastric empty, is a commonly seen complication after esophagectomy, which is not typically related to robotic surgery.

Robotic-assisted esophageal surgery has been consolidated in recent years in our center, for the treatment of malignant and benign tumors of the esophagus; in this series, we describe as the robotic procedures can be feasible and successfully applied also in the treatment of challenging cases. On the other hand, the lack of an adequate number of described cases in literature does not allow serious comparisons.

This study has of course several limitations due to the limited number of patients. Furthermore, the heterogeneity of the study population and different surgical procedures cannot lead to strong conclusions or guidelines.

## Conclusion

Tumors of the esophageal submucosa represent surgical challenges even for experienced surgeons due to their heterogeneity. Our series show that robotic surgery, performed by a specialized center, may facilitate minimally invasive surgery in the management of complex SMT of the esophagus. We believe that the treatment of esophageal SMTs should be planned individually by experienced esophageal surgeons. More studies including an adequate number of patients are needed to confirm our optimal results.

## Data Availability

The data that support the findings of this study are available from the corresponding author upon reasonable request.
